# GlobTherm, a global database on thermal tolerances for aquatic and terrestrial organisms

**DOI:** 10.1038/sdata.2018.22

**Published:** 2018-03-13

**Authors:** Joanne M. Bennett, Piero Calosi, Susana Clusella-Trullas, Brezo Martínez, Jennifer Sunday, Adam C. Algar, Miguel B. Araújo, Bradford A. Hawkins, Sally Keith, Ingolf Kühn, Carsten Rahbek, Laura Rodríguez, Alexander Singer, Fabricio Villalobos, Miguel Ángel Olalla-Tárraga, Ignacio Morales-Castilla

**Affiliations:** 1 German Centre for Integrative Biodiversity Research (iDiv) Halle-Jena-Leipzig, Germany.; 2 Institute of Biology/Geobotany & Botanical Garden, Martin Luther University Halle-Wittenberg, Halle (Saale) 06108, Germany.; 3 Département de Biologie, Chimie et Géographie, Université du Québec à Rimouski, Rimouski, QC G5L 3A1, Canada.; 4 Centre for Invasion Biology, Department of Botany and Zoology, Stellenbosch University, Private Bag X1, Matieland 7602, South Africa.; 5 Department of Biology and Geology, Physics & Inorganic Chemistry, Rey Juan Carlos University, 28933, Móstoles, Spain.; 6 Biodiversity Research Centre, University of British Columbia, Vancouver, BC, Canada.; 7 Earth to Ocean Research Group, Department of Biological Sciences, Simon Fraser University, Burnaby, Canada.; 8 School of Geography, University of Nottingham, Nottingham, NG7 2RD, UK.; 9 Department of Biogeography and Global Change, National Museum of Natural Sciences, CSIC, Calle Jose Gutierrez Abascal, 2, 28006, Madrid, Spain.; 10 Center for Macroecology, Evolution and Climate, Natural History Museum of Denmark, University of Copenhagen, Copenhagen Ø, Denmark.; 11 Centre for Biodiversity and Genetic Resources, CIBIO, University of Évora, Largo dos Colegiais, 7000 Évora, Portugal.; 12 Department of Ecology and Evolutionary Biology, University of California Irvine, CA 92697, USA.; 13 Lancaster Environment Centre, Lancaster University, Lancaster LA1 4YQ, UK.; 14 Department Community Ecology, Helmholtz Centre for Environmental Research - UFZ, Permoserstraße 15, 04318 Leipzig, Germany.; 15 Imperial College London, Silwood Park Campus, Ascot, Berkshire SL5 7PY, UK.; 16 Swedish University of Agricultural Sciences, Swedish Species Information Centre, Box 7007, SE-750 07, Uppsala, Sweden.; 17 Departamento de Ecologia, Instituto de Ciências Biológicas, Universidade Federal de Goiás, Goiânia, Goiás, Brazil.; 18 Red de Biología Evolutiva, Instituto de Ecología, A.C. México.; 19 Arnold Arboretum, Harvard University, Boston, USA.; 20 Department of Life Sciences, University of Alcalá, Alcalá de Henares, 28802, Spain.

**Keywords:** Physiology, Biogeography, Macroecology, Climate-change ecology

## Abstract

How climate affects species distributions is a longstanding question receiving renewed interest owing to the need to predict the impacts of global warming on biodiversity. Is climate change forcing species to live near their critical thermal limits? Are these limits likely to change through natural selection? These and other important questions can be addressed with models relating geographical distributions of species with climate data, but inferences made with these models are highly contingent on non-climatic factors such as biotic interactions. Improved understanding of climate change effects on species will require extensive analysis of thermal physiological traits, but such data are both scarce and scattered. To overcome current limitations, we created the GlobTherm database. The database contains experimentally derived species’ thermal tolerance data currently comprising over 2,000 species of terrestrial, freshwater, intertidal and marine multicellular algae, plants, fungi, and animals. The GlobTherm database will be maintained and curated by iDiv with the aim to keep expanding it, and enable further investigations on the effects of climate on the distribution of life on Earth.

## Background & Summary

A long-standing challenge in ecology and biogeography is to understand what generates patterns in species diversity and distributions^1^. Undertaking this challenge is of increasing importance if we are to manage the effects of global change on biodiversity^2^. The upper and lower temperature limits to performances, sublethal irreversible conditions and molecular degradation are central to determining the geographic distributions and range shifts of species under climate change^3^. Thus, thermal tolerances limits can be used to evaluate the relative contribution of macrophysiology and macroevolution to generating species diversity gradients in terrestrial, coastal, and marine realms^4^.

Inferring species’ thermal tolerance limits based on realized climatic niches can be confounded by non-physiological factors including biotic interactions, dispersal ability, and/or habitat patch size^5,6^. Studies using experimentally-derived estimates of species’ fundamental climatic niches have significantly advanced our knowledge of how species’ ranges conform to thermal tolerance limits at land and sea^7,8^ and how thermal physiological traits are asymmetrically conserved through evolution^9^. However, these studies have generally been limited in taxonomic coverage, with only one study focused on trans-realm comparisons^7^.

In order to overcome these limitations and develop unified theories and methodologies on the influence of fundamental thermal niches on the geographic distribution of diversity worldwide and across realms, a comprehensive cross-taxon and cross-realm dataset of thermal tolerance limits is urgently needed. Here we present the GlobTherm database, a large global cross-realm multi-taxon dataset comprising published experimentally-derived species’ thermal tolerances for over 2,000 species of multicellular algae, plants, fungi and animals. Experimentally-derived measures of thermal limits provide a direct estimate of relevant aspects of species’ fundamental thermal niches^10,11^. Hence, these metrics overcome many of the confounding factors associated with the currently popular but possibly flawed method of inferring species’ thermal tolerance limits from realized geographic niches^12,13^.

Thermal tolerance limits are highly relevant to key issues in the current ecological literature, including which taxa have realized niches that are closer to their upper physiological tolerances and therefore may be more vulnerable to climate change^13^. The GlobTherm dataset centralizes data-collection efforts across taxon and synthesizes it in a format ready for researchers to use in order to conduct common analyses in macroecology, macroevolution and macrophysiology. While entries describing “thermal ranges” are often available in other databases (e.g. Fishbase, Mammalbase), the estimate of thermal tolerance is often based on distributional data and is not published alongside information on the methodology used to estimate thermal tolerance. GlobTherm is unique in collating experimentally-derived thermal tolerance data, which are independent-and thus comparable-to species’ realized ranges.

## Methods

From November 2015 until October 2016, data were compiled from published experimental estimates of upper and lower temperature tolerance limits following the protocols established by Clusella-Trullas^14^. Measures of thermal tolerance that allow the greatest across taxon coverage were targeted; these included (i) critical (threshold) and (ii) lethal temperatures. (i) Critical temperatures mark the loss of key ecological functions, such as locomotion, ability to gain nutrition, or maintain basal metabolism (as per thermal neutral zone TNZ for endotherms) and are measured with critical thermal maximum (CTmax) or minimum (CTmin), and TNZ or reduced by a predefined amount (i.e. 50%, CT50). (ii) At lethal temperatures mortality occurs in whole individuals or part thereof i.e. leaf die back to a predefined percentage (commonly measured as lethal temperature 100% (LT100) or 50% measured as LT50) after a fixed duration of time. For studies in which data were presented graphically and not stated as text, values were extracted using Plot digitizer software, version 2.0^15^. Species names and taxonomy were standardized into the National Center for Biotechnology Information (NCBI) taxonomic system using ‘taxize’ package^16^ in the statistical program R^17^.

The protocol was as follows. JMB searched for published articles, books and thesis using the following search terms: ‘critical thermal maximum’, ‘critical thermal minimum’, ‘upper thermal tolerance’, ‘lower thermal tolerance’, ‘thermal tolerance breadth’, ‘heat tolerance’, ‘cold tolerance’, ‘upper lethal temperature limit’, ‘lower lethal temperature limit’, ‘thermal tolerance window’, ‘species temperature tolerance’, ‘thermo-neutral zone’, and ‘frost resistance’ in Google Scholar (see Table 1 (available online only)). JMB then examined the abstracts and methods sections of the manuscripts to determine if they complied with our selection criteria. When insufficient information on experimental methods or sampling locations was provided within the publication, the authors were contacted to request additional information. Measures of thermal tolerance were only recorded if methodology and sampling locations were provided (either in the manuscript or by the author). When reviews were found in the literature search that complied with our data quality requirmens, the cited papers or authors attributed were located and the data extracted from these original sources when possible. A total of 567 studies were found to provide data of a high enough quality to be included in the dataset, out of the thousands of candidate studies.

Species phenotypes are intrinsically plastic. In particular, thermal limits show a considerable level of plasticity among different life stages and/or populations of a same species living along temperature gradients associated with latitude. To make the estimates of species thermal limits in the dataset comparable, only estimates from study specimens in their later life stages were used, i.e. eggs, larvae, seeds, gametes etc. were all excluded from the present form of our dataset. When multiple estimates for a species’ thermal limits were available, to standardize methodologies between estimates as much as possible, priority was given to estimates that had the greater share of the following attributes with more weight given to attributes in the following order: (1) thermal limits measured using more common metrics, i.e. CTmax and CTmin over LT50, LT50 over LT100, and LT100 over super cooling point (SCP) (with the exception of mammals and birds for which all data were TNZ and algae where lethal measures were given preference due to the inconstancy among the methods used to determine critical measures in these taxa) (2) estimates of upper and lower thermal limits in the same population; (3) field-fresh specimens over acclimated specimens and acclimated specimens over those in long-term captivity; (4) whole individuals over part specimens, (i.e. tree branches); (5) measurements taken during active seasons and phases (i.e., diurnal during the day and overnight for nocturnal species); (6) measurments with larger sample sizes (7) measurements taken from fasted individuals over fed; (8) mean measures over median (due to the paucity of the latter); (9) the loss of righting response and/or locomotion over the onset of spasms (OS) as the end point of CTmax and CTmin in ectothermic animals (due to the rarity of OS); and (10) estimates with stronger supporting information including location, ramping rate (rate of temperature increase) and acclimation temperature. In all cases, these criteria lead to the selection of a single study that optimized comparability between species measures. Despite such precautions variations in the methods used between studies will add some random error to the estimates, however our methods should not bias the error in any one direction^14^.

Data were excluded if measurements were taken from individuals bred for commercial purposes, such as agriculture, aquaculture, or the pet trade, to reduce confounding issues associated with artificial selective history. Individuals held in managed populations i.e., zoos, university labatory populations and botanical gardens or those bought from wild life traders were only used if we were able to insure the animals were not of a commercial origin. If this information was not provided in the manuscript i.e. if the location of their original wild capture/collection was not given the authors were contacted before a study was included.

## Data Records

**Figure 1:**
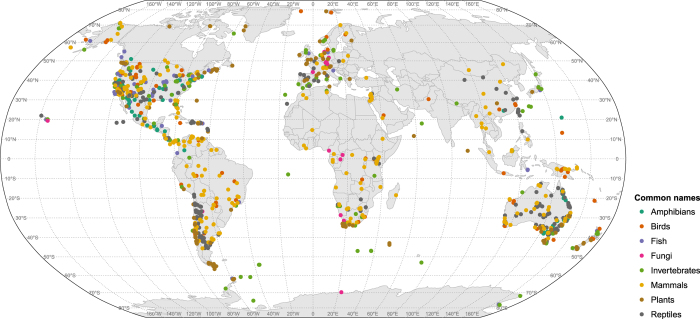
A map illustrating the geographic location at which experimental species were collected. Points are colored according to the common name in which the species belongs including fungi, plants, algae, invertebrates, fish, amphibians, reptiles, birds and mammals.

**Figure 2:**
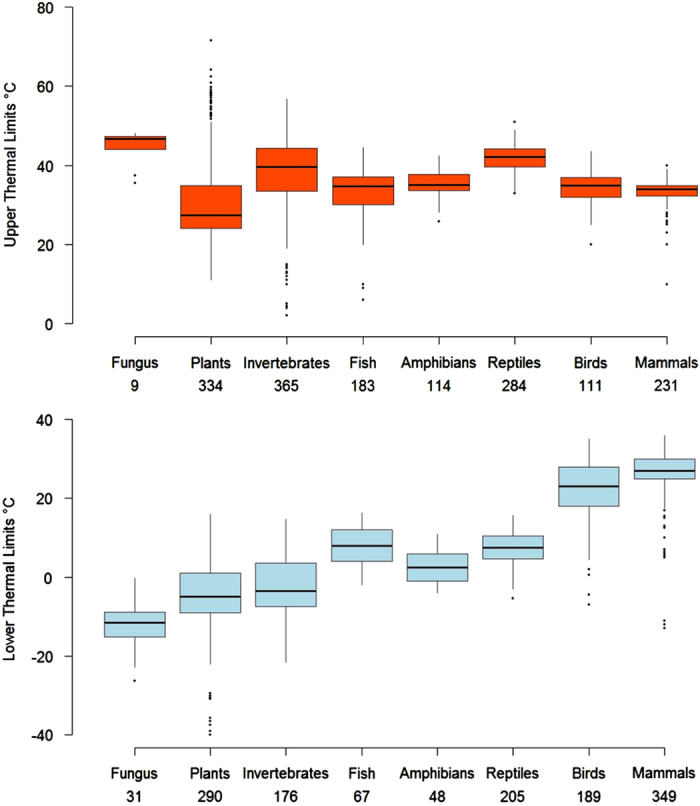
Boxplot of the mean upper and lower thermal limits for fungi, plants, algae, invertebrates, fish, amphibians, reptiles, birds and mammals, the total number of records for each group shown below. Upper thermal tolerance is shown in red and lower thermal tolerance in blue, whiskers show the maximum and minimum quartiles.

This database includes thermal tolerance metrics for 2,133 species of multicellular algae, plants, fungi, and animals in 43 classes, 203 orders and 525 families from marine, intertidal, freshwater, and terrestrial realms, extracted from published studies (Data citation 1, and Figures 1 and 2). The data presented here are available in both Excel and text formats in the Data Dryad (Data citation 1). Updates to the data and metadata will be curated through the iDiv data portal (https://idata.idiv.de/). For example, in the future it is planned to include intraspecific variation in the dataset, to provide multiple estimates of thermal tolerance limits for a given species. Where, estimates determined using the best possible methods will be more highly ranked.

## Technical Validation

JMB gathered the data from published and peer-reviewed scientific studies. The differences among experimental methods, observers, and pre-conditions (i.e. season and capture locations) are known to generate some variance in the estimates of species temperature tolerance. Information relating to experimental methods were recorded alongside the thermal tolerance limits to enable data users to incorporate these parameters in data analyses and approaches for methods validation of data. Provision of such metadata also enables users to filter data based on their specific needs and research questions.

In particular, the experimental methods used to determine the lethal temperature for algae and the upper boundary of the thermal neutral zone (UTNZ) for mammals and birds may have an effect on the quality of the estimate. We provide the temperature intervals between lethal measurements for algae and information on the quality of the regression used to estimate the UTNZ for mammals and birds (for more information on each column in the dataset please see Table 2 (available online only)). Similar to other assessments of the quality of published UTNZ measures^18–20^ we found that only ~50 % of the literature compiled contained valid estimates i.e., evidence that the boundary of the UTNZ was reached in the experiment.

The dataset has a wide global spatial coverage (Figure 1), though clear geographical data gaps do exist, for example, in central Africa, Russia, India, parts of Canada and in the deep ocean. The data gaps present in this study are unfortunately common as they represent locations that are either hard to access due to geography (i.e. northern Canada and Russia, deep ocean, the tropics), or where scientific literature is difficult to access due to language and related citation indexing barriers^21^. The distribution of the data across realms reflects the distribution of known species on Earth, where ~80% of macroscopic species live on land (most being insects) compared to 15% in the ocean (showing however the greatest phyla difference) despite the much larger area and volume, and 5 % in freshwater^22,23^. The dataset contains approximately 0.20% of plants^24^, 0.72 % of algae^25^, 0.00024 % of insects^23^, 0.55% of fish^26^, 3.33% of reptiles^23^, 6.01% of mammals^27^, 1.86% of birds^27^ currently described. Taxonomically, Chordata are over-represented in our data set, while algae, plants, and, to a greater extent, invertebrates, are underrepresented given their greater contribution to the world’s total number of species. In sum, the GlobTherm dataset reflects both geographic and taxonomic bias in sampling of thermal tolerances, which may ultimately help identifying gaps and guiding subsequent efforts in documenting the thermal physiology of life on Earth.

## Additional information

**How to cite this article**: Bennett, J. M. *et al.* GlobTherm, a global database on thermal tolerances for aquatic and terrestrial organisms. *Sci. Data* 5:180022 doi: 10.1038/sdata.2018.22 (2018).

**Publisher’s note:** Springer Nature remains neutral with regard to jurisdictional claims in published maps and institutional affiliations.

## Supplementary Material



## Figures and Tables

**Table 1:** Search terms used to select papers for the literature characterisation and data sources.

**Search Terms**	“critical thermal maximum” OR “critical thermal minimum” OR “upper thermal tolerance” OR “lower thermal tolerance” OR “thermal tolerance breadth” OR “heat tolerance” OR “cold tolerance” OR “upper lethal temperature limit” OR “lower lethal temperature limit” OR “thermal tolerance window” OR “species temperature tolerance” OR “thermo-neutral zone” OR “Frost resistance”
**Time period**	November 2014 – October 2015
**Journals**	Acta Ecologica Sinica, Acta Theriologica, Acta Zoologica Sinica, African Journal of Ecology, American Journal of Physiology, American Journal of Primatology, American Midland Naturalist, American Naturalist, Animal Behaviour, Annales Botanici Fennici, Annals of Forest Science, Applied Phycology, Aquaculture Research, Archives of Physiology and Biochemistry, Aquatic Ecology, Arctic and Alpine Research, Arctic, Antarctic, and Alpine Research, Australian Journal of Zoology, Berichte der Deutschen Botanischen Gesellschaft, Biological Conservation, Biological Journal of the Linnean Society, Biology Letters, Biotropica, Botanica Marina, British Phycological Journal, Bulletin of the AMNH, Bulletin of the Southern California Academy of Sciences, Canadian Journal of Botany, Canadian Journal of Fisheries and Aquatic Sciences, Canadian Journal of Zoology, Cell Stress and Chaperones, Chesapeake Science, Chinese Journal of Zoology, Communications in Agricultural and Applied Biological Sciences, Physiology, Comparative Biochemistry and Physiology Part A: Molecular & Integrative Physiology, The Condor, Copeia, Current Zoology, Ecological Applications, Ecological Entomology, Ecological Monographs, Ecology, Ecoscience, Ecology Letters, Ecotropicos, Environmental Biology of Fishes, Environmental Entomology, Environmental and Experimental Botany, European Journal of Phycology, Evolution, Freshwater Biology, Functional Ecology, Global Change Biology, Helgoländer Meeresuntersuchungen, Herpetologica, Hydrobiologia, Ibis, Iguana, Insectes sociaux, Integrative and Comparative Physiology, International Congress Series, Journal of Applied Physiology, Journal of Arachnology, Journal of Arid Environments, Journal of Biology, Journal of Biogeography, Journal of Cellular Physiology, Journal of Comparative Physiology A: Neuroethology, Sensory, Neural, and Behavioral Physiology, Journal of Comparative Physiology B: Biochemical, Systemic, and Environmental Physiology, Journal of Ecology, Journal of Experimental Biology, Journal of Experimental Marine Biology and Ecology, Journal of Experimental Zoology, Journal of the Fisheries Board of Canada, Journal of Freshwater Ecology, Journal of Evolutionary Biology, Journal of Herpetology, Journal of Insect Physiology, Journal of Mammalogy, Journal of the Marine Biological Association of the United Kingdom, Journal of Molluscan Studies, Journal of Phycology, Journal of Sea Research, Journal of Stress Physiology & Biochemistry, Journal of Thermal Biology, Journal of Zoology, Koedoe, Limnology and Oceanography, Marine Biology, Marine Ecology Progress Series, Marine Mammal Science, Nature, Nature Communications, Netherlands Journal of Zoology, New Zealand Journal of Marine and Freshwater Research, New Zealand Journal of Zoology, Oecologia, Ornis Scandinavica, Oikos, Philosophical Transactions of the Royal Society B: Biological Sciences, Phycologia, Physiological and Biochemical Zoology, Physiological entomology, Physiological Zoology, Planta, Plant Biology, PLoS One, Polar Biology, Proceedings of the National Academy of Sciences, Proceedings of the Royal Society of London B: Biological Sciences, Protoplasma, Restoration Ecology, Revista Brasileira de Biologia, Revista Chilena de Historia Natural, Science, Texas Journal of Science, The Auk, The Biological Bulletin, The Bryologist, The Journal of Japanese Phycology, The Southwestern Naturalist, The Wilson Bulletin, Transactions of the American Fisheries Society, Transactions of the Royal Society of South Australia, Tree Physiology, Zeitschrift für vergleichende Physiologie, Zoology, Zoological Research, Zoological Studies
For the full list of the data sources, as per the data set, with DOI when provided and information on the study specimens in each data source including the primary realm the specimens belong to and the continent on which the specimens were collected please see the refernces file included in Data citation 1.	

**Table 2:** Metadata Records for each column in the GlobTherm dataset. Metadata headings for each column in the dataset

**Column header**	**Metadata descriptor**
Genus	Genus taxonomic rank, as per NCBI.
Species	Species name, as per NCBI.
N	Total number of individuals used by the experiment. If a range was given the mid point was used.
Tmax	The upper thermal limit in degrees °C.
Error	Sample error if provided in text
Error measure	The measure of sample error i.e. standard deviation (SD), standard error (SE) etc.
Multiple measures	If both critical and lethal measures of thermal tolerance were available for a species form a source it is indicated by (Y) otherwise (N) when not available.
max_metric	The metric associated with the upper thermal limit i.e. CTmax, CT50, LT50, LT100.
max_interval_after_LT0	Only in the case of algae. Generally, thermal limits in algae are measured using water baths set at different temperatures. In some studies the temperature intervals between baths are large, which makes determining the temperature at which death occurred difficult. In these cases we recorded both LT0 (all alive) and LT100 (all dead) and the size of the interval between records i.e., 1 °C, 2 °C, 5 °C, 10 °C directly after LT0 was recorded i.e. an LT0 of 10 °C and an interval of 10 °C would indicate that all specimens were alive in a water bath of 10 °C and that the next recording was taken in a water bath 10 °C warmer (20 °C) in which some individuals were recorded as dead.
Tmax_2	Only for algae: upper lethal limit with 100% mortality (in degrees °C).
max_metric_2	Only for algae, always LT100 (100% death)
max_interval_before_LT100	Only in the case of algae. The size of the interval between experimental water baths in °C directly before all individuals were recorded as dead (LT100). i.e. an LT100 of 10 °C with an interval of 10 °C would indicate that the all specimens were dead in a water bath of 10 °C and that the previous recording was taken in a water bath at 10 °C cooler (0 °C) in which some individuals were recorded as alive.
max_pretreatment	For experiments that had acclimated individuals, the temperature at which the species were held prior to experimentation. If the species was identified as field fresh this is indicated by the letter F.
max_ramp	The rate of temperature change at which individuals were warmed in °C per minute, if given by the study.
lat_max	Latitude.
long_max	Longitude.
elevation_max	Elevation.
REF_max	Reference of the data source.
location_max	1: the location or GPS was given in the study; 2: the region or area of occurrence was given in the article, and a location from the same area or region was chosen given records for the species; 3: the location information was not given other than the specimen was wild caught. In this case the middle of the range is given when a range map for the species was available.
N	Total number of individuals used by the experiment. If a range was given the mid point was used.
tmin	The lower thermal limit in degrees C.
min_metric	The metric associated with the lower thermal limit i.e. CTmin, LT50, LT100.
Error	Sample error if provided in text
Error measure	The measure of sample error i.e. standard deviation (SD), standard error (SE) etc.
Multiple measures	If both critical and lethal measures of thermal tolerance were available for a species form a source it is indicated by (Y) otherwise (N) when not available.
min_interval_after_LT0	Only in the case of algae. The size of the intervals between measurements in °C directly after LT0 was last recorded (for further explanation see max_interval_after_LT100).
LT100	Only for algae: lower lethal limit with 100% mortality in degrees C.
min_metric_2	Only for algae, always LT100 (100% death).
min_interval_before_LT100	Only in the case of algae. The size of the intervals between measurements in °C directly before LT100 is first recorded (for further explanation see max_interval_before_LT100).
min_pretreatment	For experiments that had acclimated individuals, the temperature at which the species were held prior to experimentation. If the species was identified as field fresh this is indicated by the letter F.
ramp_min	The rate of temperature change at which individuals were cooled in °C per minute, if given by the study
lat_min	Latitude.
long_min	Longitude.
elevation_min	Elevation.
REF_min	Reference of the data source.
location_min	Same methods as those used for location_max.
Phylum	Taxonomic level as per NCBI.
Class	Taxonomic level as per NCBI.
Order	Taxonomic level as per NCBI.
Family	Taxonomic level as per NCBI.
Quality of TNZ	1: high quality estimate, conclusive evidence of an increase in metabolic rates, demonstrated by either multiple measurements beyond the (TNZ) or high intensity sampling, 2: lower quality estimate - evidence of an increase in metabolic rate but insufficient data to fit a regression to estimate the UTNZ due to either a low sample size or low sampling intensity i.e. interval larger than 1 °C between measurements, 3: Data was excluded from the dataset when no evidence of an increase in metabolic rate was presented to support the UTNZ i.e. no measurements beyond the stated TNZ in text. NA indicates where a study appeared to have appropriate methods either based on peer comms or the original manuscript but the raw data was not presented for assessing the regression used to determine the UTNZ.
